# Aggregation Tendency, Cellular Uptake, and Viability Effects of Structurally Distinct Carbazole–Phthalocyanine Gold Nanoconjugates

**DOI:** 10.1021/acsorginorgau.5c00107

**Published:** 2026-01-26

**Authors:** Neval Sevinç Özdemir, Özlem İpsiz Öney, Hacer Yasemin Yenilmez, Nazlı Farajzadeh Öztürk, Zehra Altuntaş Bayır

**Affiliations:** † Department of Pharmaceutical Basic Sciences, Faculty of Pharmacy, Acıbadem Mehmet Ali Aydınlar University, Atasehir, Istanbul 34752, Turkiye; ‡ ACU Biomaterials Center, Acıbadem Mehmet Ali Aydınlar University, 34752 Istanbul, Turkiye; § Department of Chemistry, 52971 Istanbul Technical University, Maslak, Istanbul 34469, Turkiye; ∥ Department of Analytical Chemistry, Faculty of Pharmacy, Acibadem Mehmet Ali Aydınlar University, Atasehir, Istanbul 34752, Turkiye

**Keywords:** aggregation, carbazole, cellular uptake, gold nanoparticles, phthalocyanines, viability

## Abstract

Phthalocyanine–gold nanoparticle (Pc–AuNP) conjugates combine the unique properties of gold with the therapeutic potential of phthalocyanines, offering a promising strategy for cancer therapy. Here, two novel carbazole-containing Pcs, axially disubstituted Si­(IV) and peripherally tetra-substituted Zn­(II) derivatives, were synthesized and conjugated to gold nanoparticles of two core sizes (20 and 40 nm). Characterization was performed using TEM and SEM techniques. Stability assays in complete medium showed a stronger aggregation tendency for SiPc–AuNPs than for ZnPc–AuNPs. Bright-field microscopy revealed that Pc–AuNPs induced detachment of A549 lung adenocarcinoma cells but not HUVEC endothelial cells, highlighting a cell type–dependent effect. Despite this detachment, no significant loss of viability occurred at 72 h, underscoring the resilience of A549 cells to membrane and cytoskeletal stress. Once internalized, both SiPc- and ZnPc-based nanoconjugates displayed similar cytoplasmic and perinuclear localization, suggesting uptake was dominated by the AuNP carrier. Preliminary MTT assays showed dye–particle interference, leading to use of the PrestoBlue assay, which avoids insoluble formazan artifacts. Viability analysis indicated that only **Au40/SiPc** transiently increased A549 reducing capacity at 24 h, likely due to short-term ROS scavenging, which normalized by 72 h. Overall, these findings demonstrate how metal center, substitution geometry, and particle size collectively affect aggregation, cellular interactions, and cytotoxic profiles, providing insights for optimizing Pc–AuNPs as nanophototherapeutic agents.

## Graphical abstract

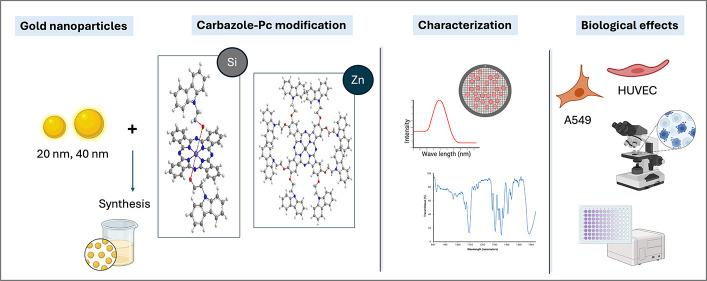

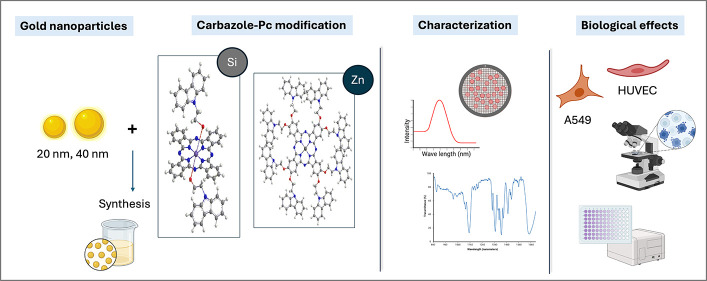

## 1 Introduction

Cancer remains one of the leading causes of mortality worldwide, driving continuous efforts to develop more effective and targeted therapeutic strategies. Despite considerable advances in surgery, chemotherapy, and radiotherapy, these conventional modalities often suffer from off-target toxicity, drug resistance, and poor selectivity, underscoring the urgent need for alternative approaches. In this context, nanotechnology has emerged as a transformative platform, offering novel opportunities for cancer diagnosis, imaging, and therapy.^1,2^

Nanomaterials in the size range of 1–100 nm possess dimensions comparable to biomolecules and cellular components, enabling unique interactions with biological systems. Their physicochemical properties facilitate passive accumulation in tumors via the enhanced permeability and retention (EPR) effect, eliminating the need for active targeting in certain cases. Among these, noble metal nanoparticles—particularly gold nanoparticles (AuNPs)—stand out due to their biocompatibility, straightforward surface modification, and tunable optical/electronic features. The surface plasmon resonance (SPR) phenomenon of AuNPs provides remarkable light absorption and scattering capabilities that can be precisely tuned through particle size, shape, surface chemistry, and the surrounding medium. Surface functionalization further improves colloidal stability, reduces aggregation, prolongs circulation time, and enables targeted delivery to desired tissues or cells.^3,4^

Phthalocyanines (Pcs) are macrocyclic compounds with strong absorption in the red-near-infrared region and the capacity to generate reactive oxygen species (ROS) upon light activation, making them powerful candidates for photodynamic therapy (PDT).^5,6^ PDT offers spatiotemporal control over cytotoxicity, as photosensitizers remain inactive until exposed to specific wavelengths of light, thus sparing healthy tissues. However, the therapeutic potential of Pcs is often hindered by their hydrophobic nature and strong tendency to aggregate in aqueous environments, which limits bioavailability, cellular uptake, and overall efficacy.^5–7^ Conjugating Pcs to AuNPs can overcome these limitations by improving dispersibility, stability, and targeted delivery while allowing fine control over photophysical behavior.^8,9^

The biological activity of Pc–AuNP conjugates is strongly influenced by the metal center and substitution pattern of the macrocycle. Zinc­(II) phthalocyanines (ZnPcs) with peripheral carbazole groups adopt a planar geometry that promotes π–π stacking and hydrophobic interactions with lipid bilayers, facilitating membrane association and cellular entry.^10–12^ In contrast, silicon­(IV) phthalocyanines (SiPcs) with bulky axial carbazole substituents possess a more three-dimensional configuration that can reduce aggregation but may limit direct membrane interaction.^13^ Carbazole moieties themselves are pharmacologically relevant, with several anticancer drugs—such as ellipticine and alectinib—featuring carbazole cores.^14^ In addition to their structural role, carbazole moieties can influence the photophysical and biological behavior of phthalocyanines. Their highly conjugated aromatic structure supports strong π–π* transitions, thereby enhancing light absorption and influencing triplet-state formation.^13,15,16^ Furthermore, amphiphilic carbazole groups can facilitate interactions with lipid bilayers and enhance nanoparticle membrane penetration.^17^ Therefore, carbazole groups are potentially bioactive determinants that can shape both colloidal behavior and biological effect of the nanoconjugates (NCs) in which they are incorporated.

Lung cancer, particularly nonsmall cell lung cancer (NSCLC), remains one of the most prevalent and deadly malignancies globally.^18^ The A549 human lung adenocarcinoma cell line is a widely accepted in vitro NSCLC model for evaluating cytotoxicity, drug uptake, and phototherapeutic outcomes.^19^ To assess selectivity and minimize off-target concerns, human umbilical vein endothelial cells (HUVECs) are commonly used as a representative healthy cell model in nanotoxicology studies.^20,21^ Despite numerous studies on AuNP-based delivery systems and phthalocyanine photosensitizers, systematic investigations addressing how variations in metal center, substitution geometry, and nanoparticle size collectively influence aggregation, cellular interaction, and therapeutic performance remain limited. Moreover, the comparative evaluation of such nanoconjugates in both malignant and healthy cell types under identical conditions is largely underexplored.

The MTT assay (3-(4,5-dimethylthiazol-2-yl)-2,5-diphenyl-2H-tetrazolium bromide) is one of the most widely used methods for testing the effects of nanoconjugates in cell viability.^22–25^ It relies on the reduction of tetrazolium into insoluble formazan crystals by the act of cellular metabolism; however, formazan potentially undergoes nanoparticle-induced redox alterations, producing artificial decreases in absorbance that are unrelated to cell viability. This interference has been increasingly reported^26–30^ and was also evident in our preliminary studies, particularly for Pc-containing Au nanoconjugates, where the formazan signal rapidly faded after measurement. To avoid such artifacts, we employed the PrestoBlue assay, which uses soluble resazurin/resorufin chemistry and reports cellular reducing capacity and thereby viability without forming insoluble products.^31,32^

In this study, we synthesized and characterized a series of Pc–AuNP nanoconjugates differing in metal center (Si­(IV) vs Zn­(II)), substitution geometry (axial vs peripheral), and particle size (20 nm vs 40 nm). Structural and morphological analyses were performed using microscopic and spectroscopic methods. Biological evaluations included dark cytotoxicity in A549 cells, biocompatibility in HUVECs, and microscopic observation of particle localization. Two novel carbazole-containing metal Pcs were synthesized and conjugated to AuNPs of distinct sizes to investigate potential synergistic effects of nanoparticle size, metal coordination, and substituent configuration. Hypothetically, bulky, axially substituted Si­(IV) phthalocyanines may exhibit reduced aggregation and more homogeneous dispersion, but weaker membrane interactions, leading to lower cellular uptake, reduced dark cytotoxicity, and potentially more controlled photodynamic therapy (PDT) responses. In contrast, planar, peripherally substituted Zn­(II) phthalocyanines are expected to display stronger membrane interactions and higher cellular uptake, resulting in more pronounced cytotoxic effects under both dark and light conditions, particularly in malignant epithelial (A549) cells. As demonstrated in other nanoparticle systems, without active targeting, Zn­(II)–phthalocyanines with optimized substitution geometry displayed higher photodynamic efficacy due to improved singlet oxygen generation and intrinsic cellular uptake.^33^ Due to their higher membrane permeability and distinct endocytic profiles, A549 cells are anticipated to internalize ZnPc–AuNPs more efficiently, whereas healthy endothelial (HUVEC) cells, with more robust barrier properties, may interact less with these nanoconjugates and thus show greater compatibility with SiPc–AuNPs. We further acknowledge that aggregation is not the sole determinant of biological performance—surface charge (zeta potential), AuNP size, and surface ligand density can substantially influence uptake and cytotoxicity. Additionally, the AuNP carrier may alter internalization kinetics, meaning that the intrinsic behavior of free Pcs may not be fully retained upon conjugation. By integrating structural, physicochemical, and biological evaluations, this work aims to provide new insights into the structure–activity relationships governing Pc–AuNP nanoconjugates as targeted nanophototherapeutic agents.

## 2 Result and Discussion

### 2.1 Characteristics of *Pc* Derivatives

Scheme 1 portrays the synthetic procedure for the newly synthesized disubstituted phthalonitrile derivative and compounds (**SiPc** and **ZnPc**). Axially disubstituted-silicon­(IV) phthalocyanine (**SiPc**) was prepared by the replacement of 9H-carbazole-9-ethanoxy groups with chlorine atoms. 4,6-bis­(9H-carbazole-9-ethoxy)­phthalonitrile was synthesized by the replacement of chlorine atoms of 4,5-dichlorophthalonitrile with hydroxyl groups of 9H-carbazole-9-ethanol via an aromatic substitution reaction. Characterization of the newly synthesized compound was carried out using ^1^H NMR, and ^13^C NMR spectroscopic techniques. Cyclotetramerization of the resultant phthalonitrile in the presence of zinc­(II) acetate in a basic medium resulted in metal phthalocyanine (**ZnPc**). The phthalocyanine derivatives were characterized by performing ^1^H NMR, UV–vis, and MALDI-TOF spectroscopic techniques.

**Scheme 1:**
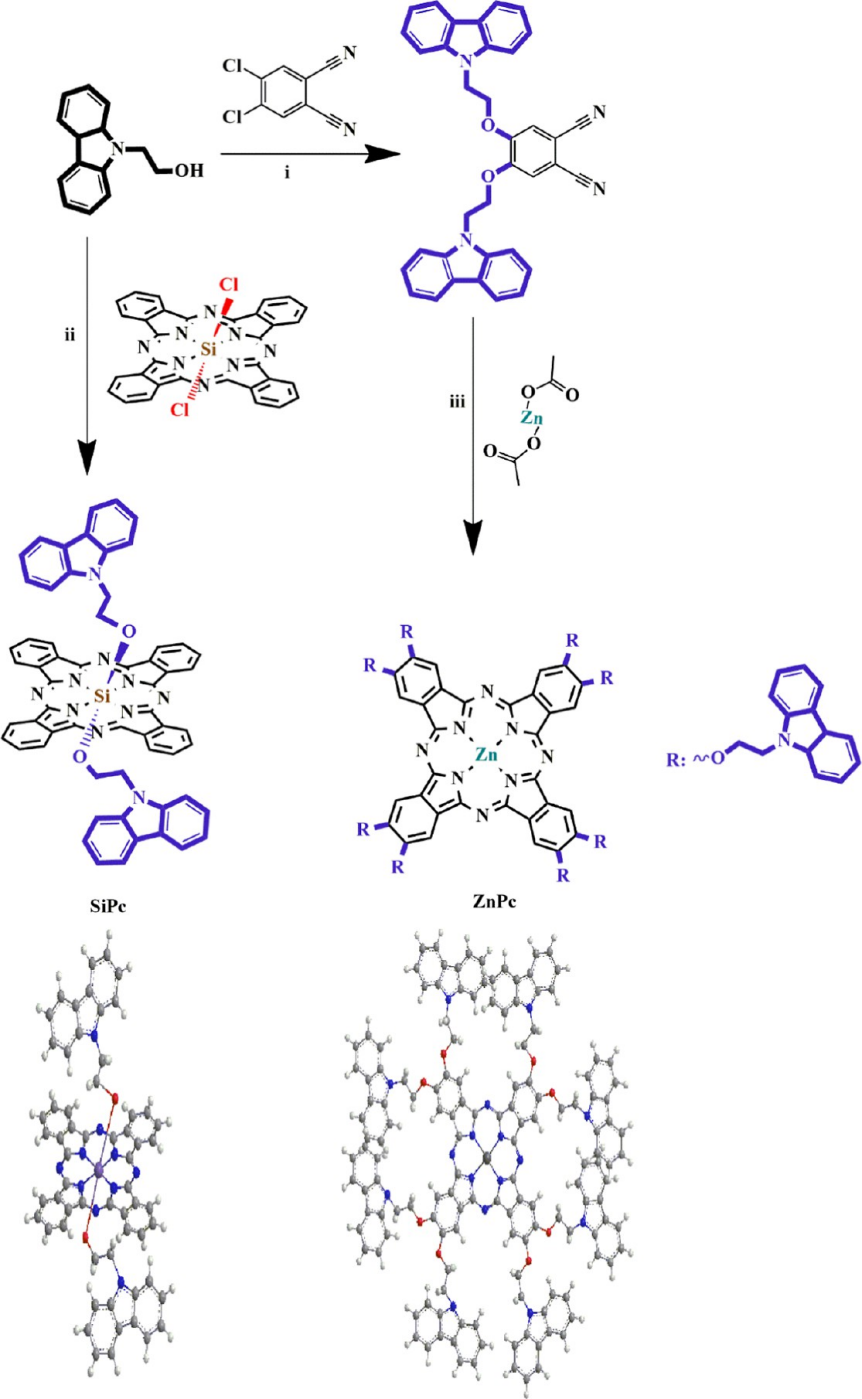
Synthetic Routes for 4,6-bis­(9H-carbazole-9-ethoxy)­phthalonitrile and Compounds (**SiPc** and **ZnPc**); (i) dry DMF, at Room Temperature, 7 days; (ii) *n*-Hexanol, DBU, 150 °C; (iii) Sodium Hydride, Toluene, 130 °C

Gold nanoparticles were prepared in two different sizes and modified with the obtained phthalocyanines through nonbonding interactions (Figure 1). Table 1 summarizes the structural characteristics of the particles with the corresponding coding. The nanoconjugates were characterized using TEM, SEM, zeta potential, FT-IR spectroscopic, UV–vis spectroscopic approaches. Some results are shown in (Figures 2–4), as examples. Figure 2 demonstrates unmodified gold nanoparticles (**Au20**) in the approximate size of 20 nm and silicon­(IV)-modified gold nanoparticles (**Au20/SiPc**). The successful synthesis of gold nanoparticles (**Au40**) in the approximate size of 40 nm and their modification with the zinc­(II) phthalocyanine (**ZnPc**) are depicted in Figure 3. The interaction of gold nanoparticles with the phthalocyanines was controlled by van der Waals forces, coordination interactions, and π–π stacking between the macromolecules and the metal nanoparticles.^34^ Additionally, the SEM images of the unmodified and modified gold nanoparticles are demonstrated in Figure 4. The surface modification of gold nanoparticles with phthalocyanine (**ZnPc**) resulted in significant differences in the SEM images of gold nanoparticles (**Au40**). The related images proved the surficial coverage of gold nanoparticles with the phthalocyanines (**SiPc** or **ZnPc**).^35^ The respective zeta potentials of unmodified gold nanoparticles (**Au20** and **Au40**) were obtained −42.3 ± 3.1 and −16.8 ± 0.8 mV. In contrast, those of the modified gold nanoparticles (**Au20/SiPc**, **Au20/ZnPc**, **Au40/SiPc**, and **Au40/ZnPc**) were obtained −1.1 ± 0.2, −0.3 ± 0.2, −8.8 ± 3.3 mV, and −8.1 ± 3.3 mV, respectively. The zeta potentials of unmodified gold nanoparticles covered with negatively charged citrate groups were negative and inversely proportional to the nanoparticles’ size. As the surface of gold nanoparticles was modified with **SiPc** or **ZnPc**, the related zeta potential values increased owing to nonbonding interactions.^35^ In the FT-IR spectra of the modified gold nanoparticles, the C=O peaks appeared around 1700 cm^–1^ and decreased by linking the phthalocyanines to the surface of gold nanoparticles via nonbonding interactions. Moreover, the characteristic bands of the phthalocyanines were observed in the FT-IR spectra of the nanoconjugates. The FT-IR spectra of unmodified gold nanoparticles and nanoconjugates are demonstrated in the Supporting Information. In the UV–vis spectra of unmodified gold nanoparticles (**Au20** and **Au40**), the SPR bands were observed at 520 and 524 nm, respectively. The UV–vis spectra of modified gold nanoparticles (**Au20/SiPc** and **Au20/ZnPc**) included the characteristic band of **Au20** and Q-bands of the phthalocyanines around 700 nm without any sharp changes. However, the UV–vis spectra of modified gold nanoparticles (**Au40/SiPc** and **Au40/ZnPc**) changed significantly with the splitting and shift of the Q-bands to the blue region.^35^

**Figure 1:**
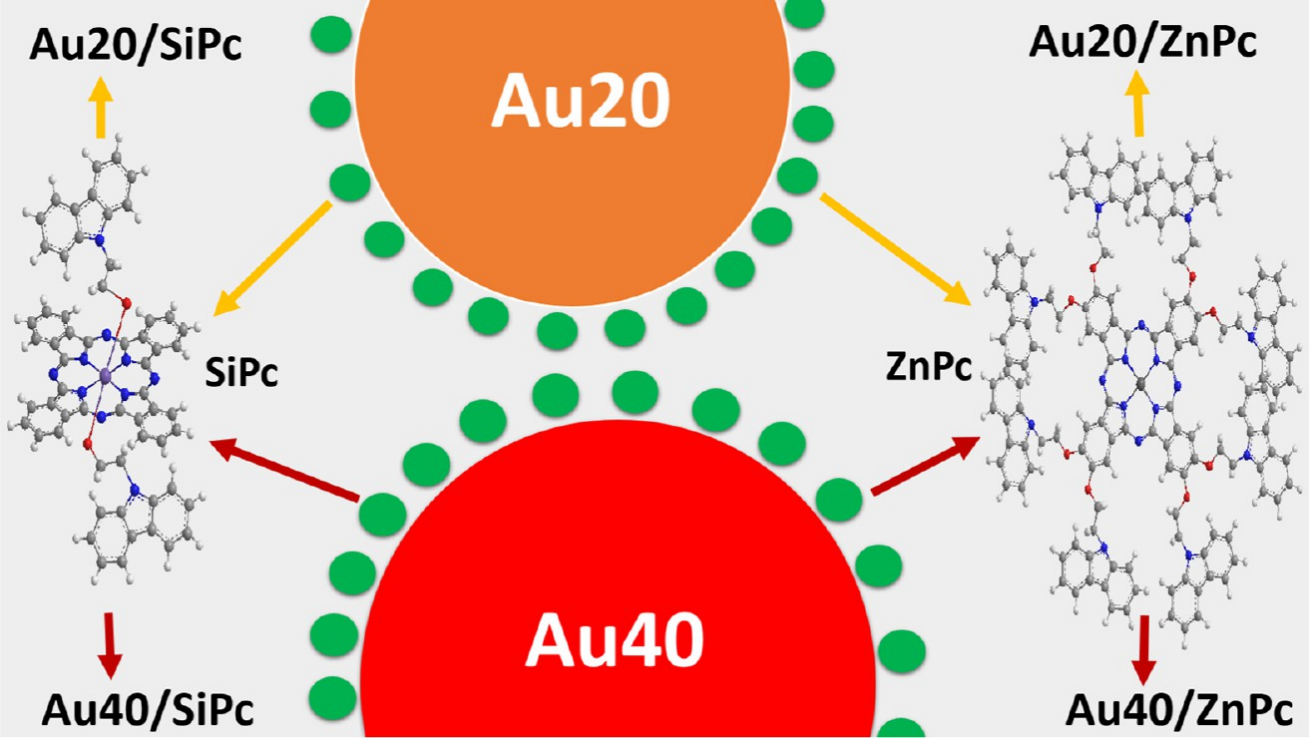
Unmodified gold nanoparticles (**Au20** and **Au40**) and phthalocyanine-modified gold nanoparticles (**Au20/SiPc**, **Au20/ZnPc**, **Au40/SiPc**, and **Au40/ZnPc**).

**Table 1:** Summary of Structural Characteristics of the Nanoconjugates Used in This Study^a^,^b^

nanoconjugate code	AuNP size	phthalocyanine type	metal core	substitution pattern	no of carbazole units	structural behavior
Au20	20 nm	none (unmodified AuNP)			0	control group
Au20/SiPc	20 nm	Si(IV)-*Pc*	Si(IV)	axial substitution	2	bulky, axial geometry
Au20/ZnPc	20 nm	Zn(II)-*Pc*	Zn(II)	peripheral substitution	4	planar, membrane-interactive
Au40	40 nm	none (unmodified AuNP)			0	control group
Au40/SiPc	40 nm	Si(IV)-*Pc*	Si(IV)	axial substitution	2	bulky, axial geometry
Au40/ZnPc	40 nm	Zn(II)-*Pc*	Zn(II)	peripheral substitution	4	planar, membrane-interactive

a Gold nanoparticles (20 and 40 nm) were functionalized with either Zn­(II)- or Si­(IV)-phthalocyanines bearing peripheral or axial carbazole groups, respectively.

b The table shows differences in size, metal core, substitution pattern, and number of carbazole units.

**Figure 2:**
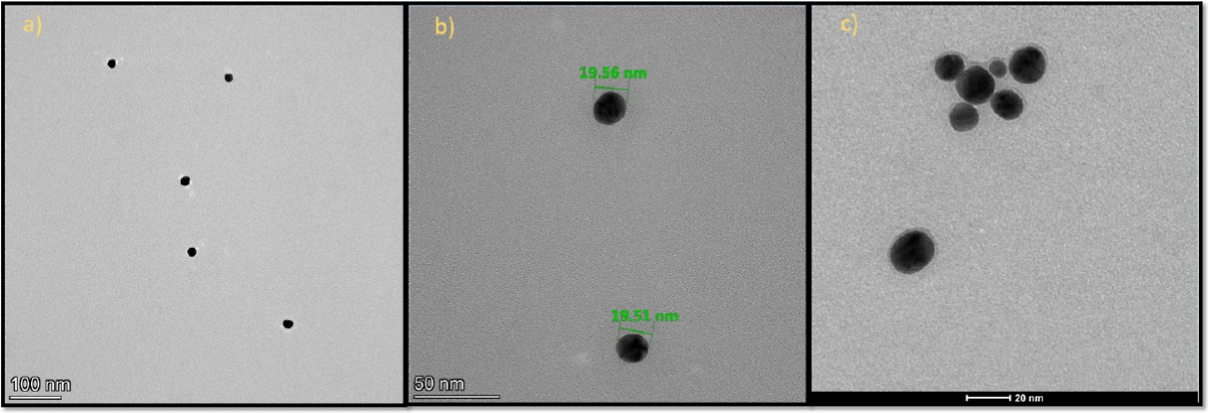
TEM images of (a,b) unmodified gold nanoparticles (**Au20**) and (c) modified gold nanoparticles (**Au20/SiPc**).

**Figure 3:**
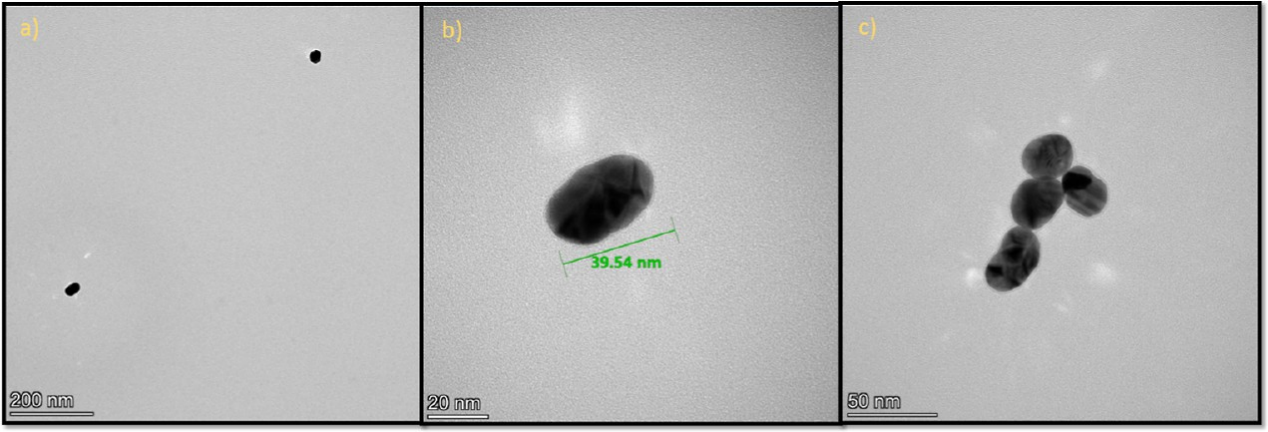
TEM images of (a,b) unmodified gold nanoparticles (**Au40**) and (c) modified gold nanoparticles (**Au40/ZnPc**).

**Figure 4:**
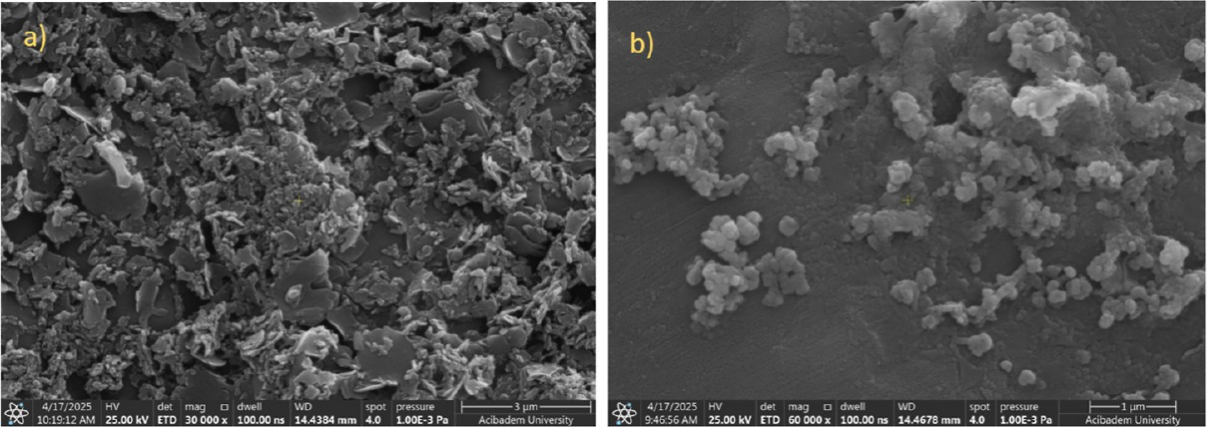
SEM images of (a) unmodified gold nanoparticles (**Au40**) and (c) modified gold nanoparticles (**Au40/ZnPc**).

### 2.2 Aggregation Tendency of NCs and Their Effect on the Morphology of HUVECs and A549 Cells

Evaluation of nanoconjugate behavior in culture medium showed that **Au20/SiPc** nanoconjugates had a stronger tendency to form visible particulates compared to their **ZnPc** counterparts which remained more evenly dispersed (Figure 5). This observation was prominent in **Au20/SiPc** containing media while **Au40/SiPc** appeared relatively more dispersed in the same conditions (Figure 6). This behavior of **Au/SiPc** nanoconjugates partially contradicts our initial hypothesis, which predicted that the bulky, axially substituted Si­(IV) derivatives would suppress π–π stacking and therefore aggregate less than the planar as also shown before.^9,36^ Instead, **Au/SiPc** displayed more visible aggregation in culture medium, while **Au/ZnPc** maintained better dispersion (Figures 5 and 6). This may stem from the increased out-of-plane hydrophobic surface area created by the axial carbazole groups, which can encourage interparticle association and protein-mediated bridging in serum-containing media.^37^ This effect could be further enhanced by a less negative surface charge, reducing electrostatic repulsion, and by the higher surface density or outward orientation of carbazole moieties, which may facilitate multivalent π–π interactions between particles, making aggregates more prominent under bright-field imaging. Additionally, electrostatic stabilization alone is not sufficient in biological media as the aggregation behavior could be sharply changed by the media composition.^38–40^ As extensively discussed in protein-corona reviews, serum proteins can either stabilize or destabilize nanoparticle suspensions depending on their abundance, binding orientation, and charge effects.^41,42^

**Figure 5:**
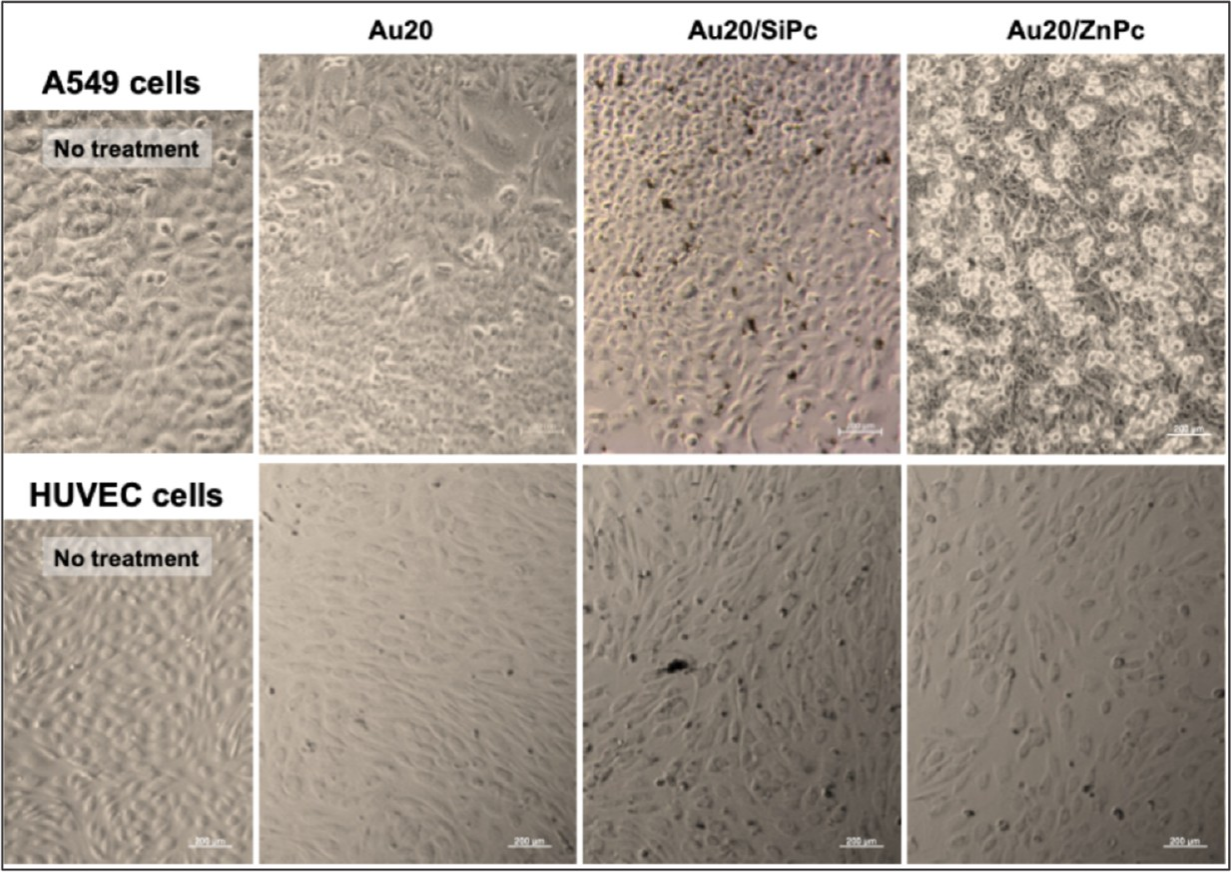
Bright field micrographs of A549 cells and HUVECs treated with **Au20** (20 nm AuNP), **Au20/SiPc**, and **Au20/ZnPc** for 24 h (37 °C, 5% CO_2_). Black dots show the aggregates in the media. Scale bar: 200 μm.

**Figure 6:**
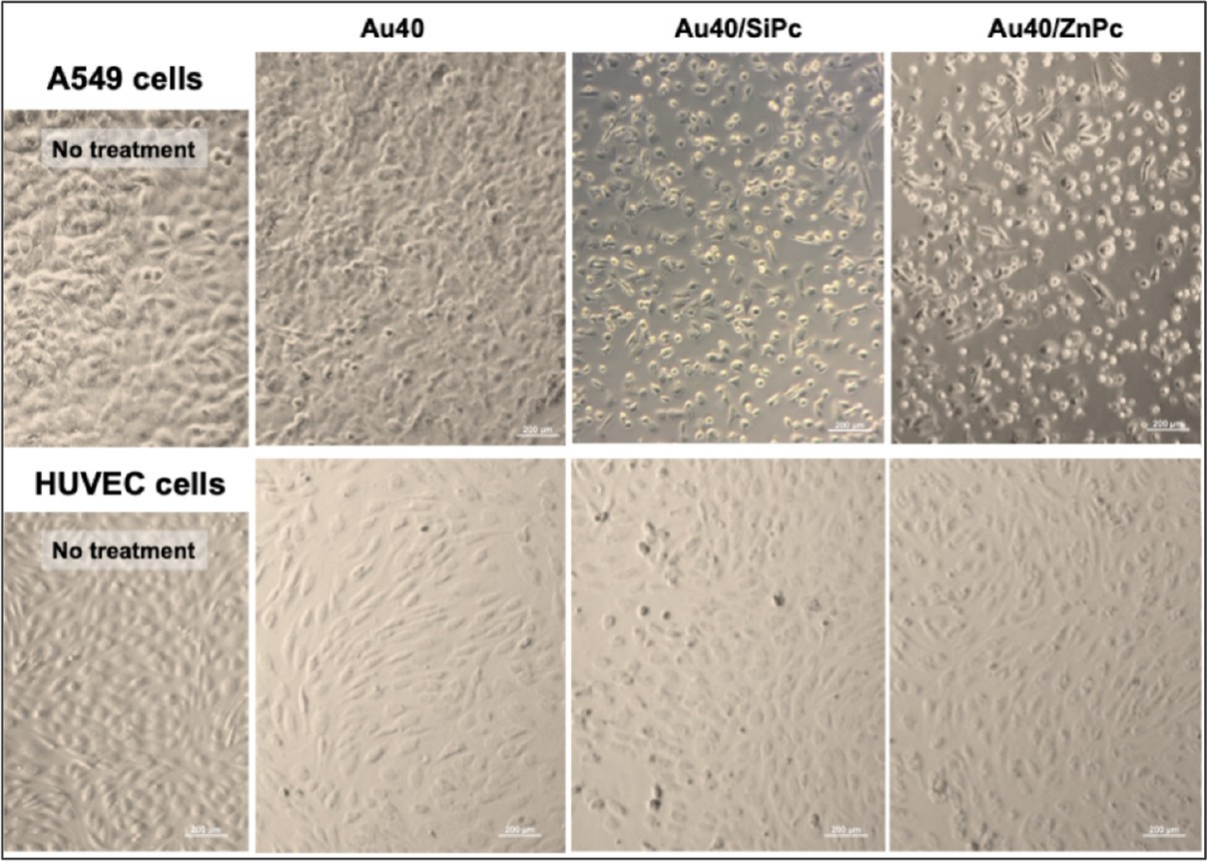
Bright field micrographs of A549 cells and HUVECs treated with **Au40** (40 nm AuNP), **Au40/SiPc**, and **Au40/ZnPc** for 24 h (37 °C, 5% CO_2_). Scale bar: 200 μm.

Bright-field micrographs revealed that all the prepared nanoconjugates, regardless of the AuNP core size, induced a pronounced detachment effect in A549 cells, as evidenced by the increased presence of circular, nonadherent cell clusters in the culture medium (Figures 5 and 6). Strikingly, this phenomenon was absent in HUVECs under identical conditions, highlighting a cell type–dependent response. The selective detachment effect in A549 cells may be linked to differences in cell adhesion properties and cytoskeletal dynamics between malignant epithelial and healthy endothelial cells. Cancer cells, such as A549, often exhibit weaker focal adhesions and altered integrin expression, making them more susceptible to detachment upon membrane perturbation or cytoskeletal stress.^43^ Pc–AuNP conjugation could increase these effects by increasing local membrane interaction, reactive oxygen species (ROS) production, or endocytic activity, even under dark conditions, leading to partial loss of substrate attachment. In contrast, HUVECs maintain stronger intercellular junctions and substrate adhesion,^44^ potentially conferring resistance to such detachment stimuli. Consistent with the previous reports, the unmodified AuNPs of both sizes (**Au20** and **Au40**) did not induce visible detachment in either cell type, nor did they reduced their viability (Figure 9).^45^ This indicates that the Pc moieties are primarily responsible for the observed effects, rather than the gold core. This is likely through their hydrophobic domains, charge distribution, or interaction with membrane proteins that regulate surface adhesion. Here another factor to consider is the physical exposure of cells to large aggregates, which is amplified by gravitational settling in 2D static cultures.^46^ The substantial size and altered surface topology of these aggregates potentially impose mechanical stress on the plasma membrane and promote lysosomal membrane permeabilization.^47,48^ Using a continuous plate rotation during incubation could help minimize sedimentation and provide a more representative assessment of nanoparticle–cell interactions.^49,50^ Nonetheless, this approach raises comparability concerns, as most studies in the field still rely on conventional static 2D culture conditions.

**Figure 7:**
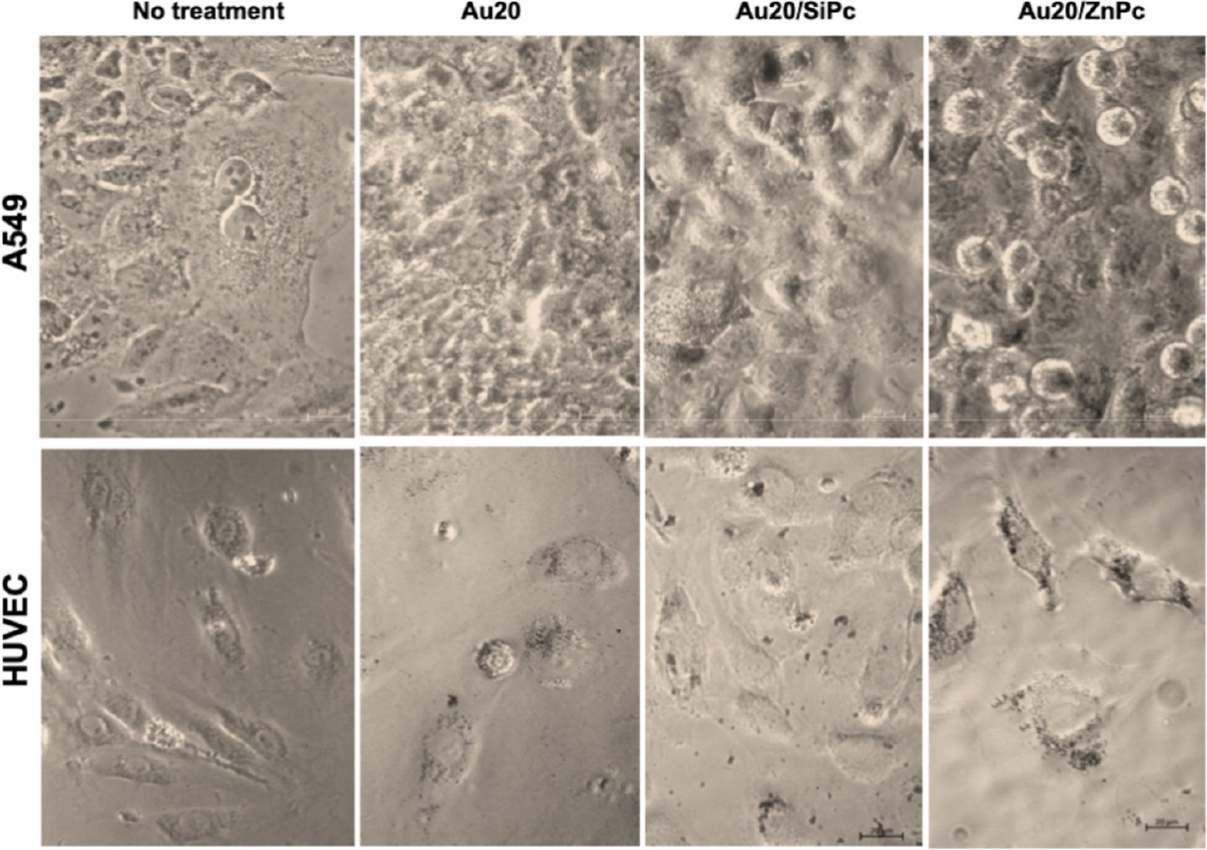
Bright field micrographs of A549 cells and HUVECs treated with **Au20** (20 nm AuNP), **Au20/SiPc**, and **Au20/ZnPc** for 24 h (37 °C, 5% CO_2_). Cellular vesicles appear as clear, bubble-like structures in the no treatment groups. Black dots indicate the intracellular accumulated particles in the perinuclear area. Scale bar: 20 μm.

**Figure 8:**
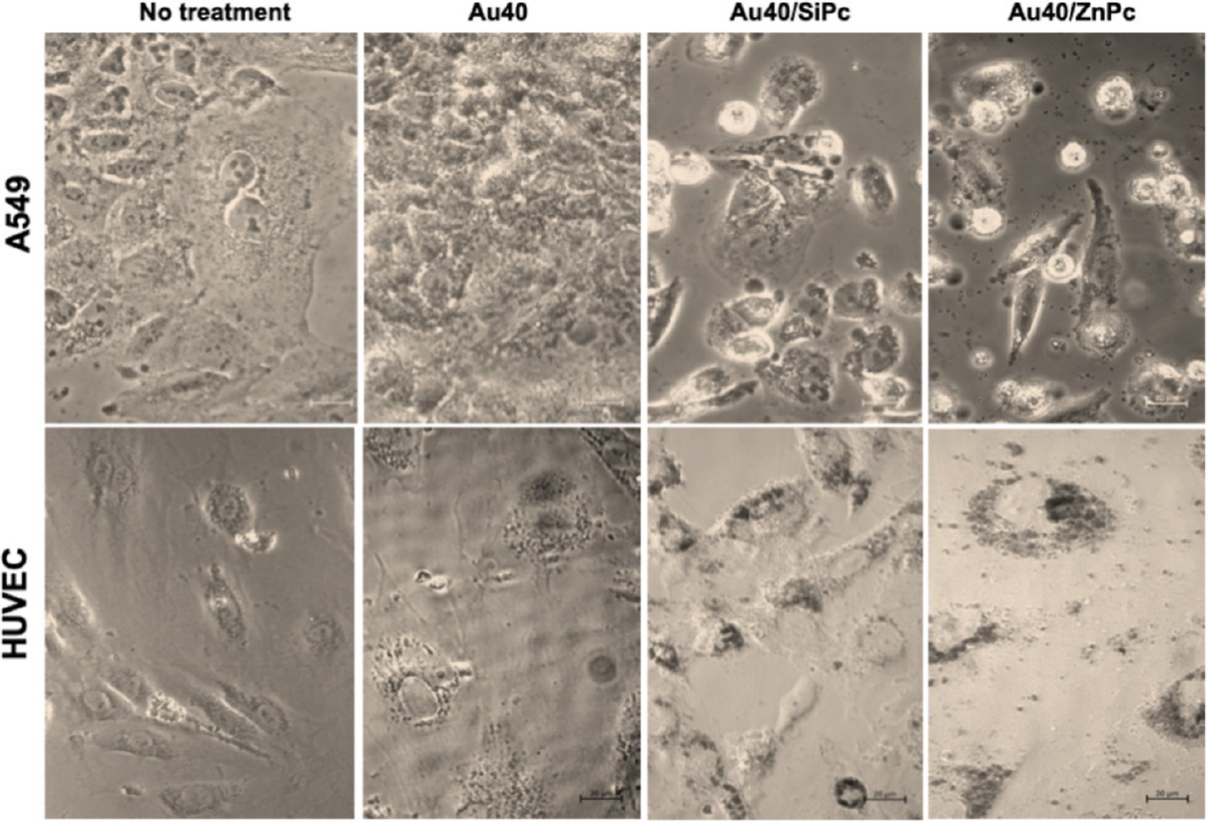
Bright field micrographs of A549 cells and HUVECs treated with **Au40** (40 nm AuNP), **Au40/SiPc**, and **Au40/ZnPc** NCs for 24 h (37 °C, 5% CO_2_). Cellular vesicles appear as clear, bubble-like structures in the no treatment groups. Black dots indicate the intracellular clusters in the perinuclear area. Scale bar: 20 μm.

**Figure 9:**
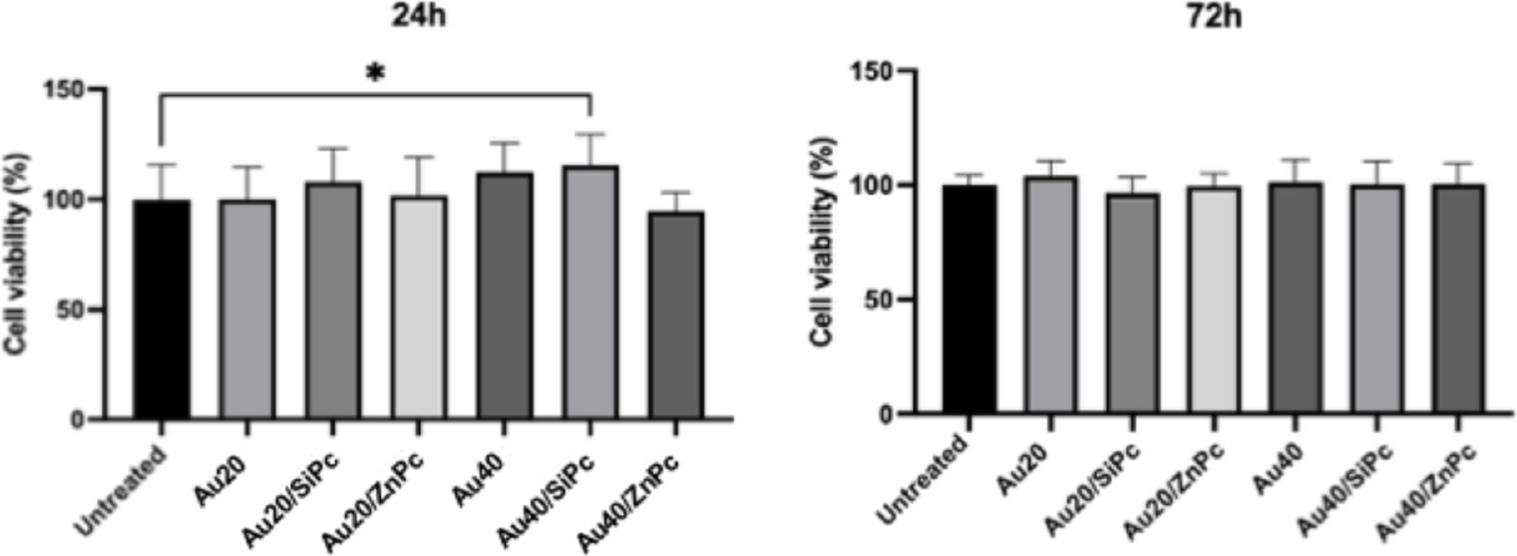
Cell viability (%) of A549 cells following treatment with nanoconjugates for 24 and 72 h. A549 cells were treated with nanostructures (**Au20**, **Au20/SiPc**, **Au20/ZnPc**, **Au40**, **Au40/SiPc**, **Au40/SiPc**; 1 μg/mL) or left untreated (control). Cell viability was assessed using the Presto Blue assay. Values were normalized to the untreated control group (set as 100%). *p < 0.05.

Furthermore, although pronounced detachment was observed in A549 cells treated with Pc-nanoconjugates, their viability at 72 h showed no significant reduction (Figure 9). This contrast is particularly relevant when comparing malignant A549 cells, which often more resilient to membrane stress due to their altered cytoskeletal and metabolic characteristics,^51,52^ with healthy endothelial cells such as HUVECs, which may be more vulnerable to acute membrane disruption under nanoparticle exposures.^53^ These observations underscore the importance of employing 3D tissue models, where cells reside in a more physiologically relevant microenvironment that better resembles native tissue architecture than conventional tissue-culture-treated plastic (TCP) surfaces, thereby improving the predictive value of in vitro findings for clinical translation.^54,55^

Au nanoparticles in the range of 10–50 nm are generally reported to be efficiently internalized by most cell types, primarily via endocytosis. However, the uptake efficiency depends on cell type, surface chemistry, particle shape, and coating.^56,57^ In our study, even though the aggregates were visible in the culture medium, the presence of internalized and clustered particles in both the **SiPc**- and **ZnPc**-treated groups can be explained by particle-size heterogeneity. Consistently, no marked difference was observed between the 20 and 40 nm AuNP groups, suggesting that heterogeneity in the effective particle population may mask size-dependent uptake differences (Figures 7 and 8). When compared with the cells that were exposed to AuNP alone, AuNP/Pc-treated groups showed more prominent intracellular clusters. This indicates that the nanoconjugates may also undergo secondary aggregation after internalization, likely within endocytic vesicles. This interpretation aligns with previous reports showing that AuNPs, although dispersed outside the cell, can form clusters following sequential trafficking through early endosomes, late endosomes, and lysosomes, particularly in the perinuclear region as previously shown.^58^

It is important to note that the interpretations based on bright field microscopy images are inherently limited, as this technique does not allow direct visualization of subcellular vesicles. Nevertheless, comparing our observations with established literature provides a useful framework for understanding the potential biological consequences of nanoparticle exposure, particularly for researchers who lack access to advanced imaging systems. In many nanomaterial studies, cytotoxicity assays are performed in isolation, while the presence of large aggregates and their mechanical or sedimentation-driven effects on cells are often overlooked. Our findings, additionally summarized in Table 2, emphasize the need for greater awareness of these limitations when evaluating the biological impact of nanoconjugates, and highlight that careful interpretation of the bright field observations, while constrained, can still offer meaningful insights when contextualized with known nanoparticle behavior.

**Table 2:** Summary of the Findings, Observations, Possible Causes, and Considerations

finding	observations/results	possible causes	limitations/notes
SiPc–AuNP nanoconjugates showed stronger aggregation while ZnPc–AuNPs maintain better dispersion	SiPc-modified AuNPs formed visible aggregates; ZnPc groups remain more dispersed and homogeneous in culture media	axial carbazole groups in SiPc may increase hydrophobic surface and π–π interactions. Planar *Pc* core in ZnPcs may result in fewer hydrophobic protrusions	static 2D culture exaggerates aggregation. Sedimentation increases particle–cell contact artificially. Medium ingredients, pH and incubation condition effects not isolated
PC-AuNP nanoconjugates caused detachment in A549 cancer cells but not in healthy HUVECs	A549 cells form nonadherent clusters; endothelial monolayers of HUVECs remain attached. However, detachment did not reduce A549 metabolic readout	weaker focal adhesions in malignant cells; Pc–AuNP membrane interactions, ROS, endocytic load. Strong VE-Cadherin junctions in HUVECs	2D plastic surfaces alter adhesion behavior. 3D culture models better reflect cellular microenvironment and shear-stress physiology
sedimentation increases nanoparticle–cell contact in static culture	large aggregates sink and interact excessively with cells. Physical collision and pressure from large aggregates possibly cause mechanical membrane stress	gravity-driven sedimentation. Aggregation-induced lysosomal stress and membrane disruption	Aggregate size not quantified. Not comparable to flow or dynamic cultures. Perfusion/rotation culture would yield different exposure profiles
unmodified AuNPs did not induce detachment in both cell types	gold cores alone do not cause adhesion loss	effect driven by *Pc* moieties	Protein corona effect should be analyzed. Corona differences may alter cellular response
nanoconjugates showed similar intracellular localization. Particles were not observable in cell nuclei	all groups showed cytoplasmic and perinuclear localization. Nuclear localization was not observed	the size of intracellular aggregates determined the internalization pattern. Nuclear pores block entry of >40 nm	As aggregation behavior varies uptake rates, dose heterogeneity affects interpretation. No single-cell quantification. TEM or super-resolution imaging is required. Localization remains partly inferred from low-resolution data
MTT formazan signal disappeared in nanoconjugate-treated wells but not in AuNP treated or untreated controls	purple MTT formazan turns transparent within ∼15 min	PCs may interact with insoluble MTT formazan, leading optical artifacts that mimic reduced viability	MTT assay is incompatible with *Pc* conjugate cytotoxicity tests in this study. PrestoBlue was chosen due to soluble rezasurin/resofurin reaction, and confirmed with the stability of the obtained color in each test groups
Au40/SiPc showed increased viability at 24 h in PrestoBlue. Twenty-four h redox effect disappears at 72 h	higher % viability vs control (*p* < 0.05) was observed in Au40/SiPc treated cells. All groups turned to control levels at 72 h	likely reflects reduced intracellular ROS → higher NAD(P)H signal at 24 h by metabolic adaptation or re-equilibration	redox kinetics was not measured; ROS assays are required for confirmation

### 2.3 The Effect of NCs on A549 Cell Viability

During preliminary cytotoxicity experiments by applying the widely used MTT reagent, we observed that in wells containing Au/SiPc and Au/ZnPc nanoconjugates, the characteristic purple formazan color faded within approximately 15 min after the absorbance reading, ultimately becoming transparent. This suggested a potential interference between the nanoparticles and the formazan product, possibly through adsorption onto the particle surface—phenomena previously reported for various nanomaterials including gold and silver particles.^26,59^ Such interactions can result in artifactual decreases in optical density unrelated to actual cell viability. Given this concern, we employed the PrestoBlue assay, which relies on soluble resazurin/resorufin chemistry and is less susceptible to particle–dye interactions.^60^ PrestoBlue measurements across seven treatment groups at 24 and 72 h revealed a single significant deviation at 24 h: **Au40/SiPc** displayed higher % viability compared to the untreated group (*p* < 0.05), whereas all groups converged to control-like levels by 72 h (Figure 9). Because PrestoBlue reports the cellular reducing capacity (enzymatic conversion of resazurin to resorufin via NAD­(P)­H-dependent mitochondrial and cytosolic reductases),^61^ the transient increase at 24 h likely reflects a temporary shift toward a more reduced intracellular redox state rather than genuine hyperproliferation. While the precise cause remains to be confirmed, a plausible explanation is that **Au40/SiPc** reduced intracellular ROS levels under dark conditions, thereby alleviating oxidative stress and increasing the NAD­(P)­H/resorufin signal (an antioxidant-type response). Future work should include quantitative ROS assays (e.g., DCFDA fluorescence) and complementary redox/mitochondrial activity measurements to validate this interpretation. By 72 h, this effect had subsided—likely due to cellular adaptation, changes in protein corona/dispersion dynamics, or metabolic re-equilibration—resulting in values approaching those of the untreated control.

## 3 Materials and Methods

### 3.1 Synthesis and Characterization

#### 3.1.1 Silicon­(IV) Phthalocyanine (**SiPc**)

Silicon­(IV) phthalocyanine dichloride (0.100 g, 0.163 mmol) and 9H-carbazole-9-ethanol (0.069 g, 0.327 mmol), and sodium hydride (0.020 g, 0.818 mmol) were refluxed in toluene (2 mL) for 24 h under an inert atmosphere. The reaction was carried out in an oil bath. After cooling to room temperature, the solvent was removed by evaporation. The crude product was purified using a column chromatographic technique (alumina: stationary phase; tetrahydrofuran: mobile phase). A dark bluish-green powder was collected after evaporation of the mobile phase. Yield: 0.082 g (52%), mp >250 °C. ^1^H NMR (500 MHz; DMSO-*d*_6_): δ 8.31–8.29 (m, 4H), 7.93–7.91 (bs, 4H), 7.81–7.78 (d, 8H), 7.57–7.51 (t, 8H), 7.48–7.43 (m, 8H), 0.85–0.82 (m, 4H), −0.06-(−0.09) (bs, 4H). UV–vis (DMSO), λ_max_, nm: 345, 684. MS (MALDI-TOF): *m*/*z* calcd for C_60_H_40_N_10_O_2_Si [M]^+^, 961.11; found, 960.83 [M]^+^, 1264.01 [M+2DHB-5H]^+^.

#### 3.1.2 4,5-bis­(9H-carbazole-9-ethoxy)­phthalonitrile

4,5-dichlorophthalonitrile (1.0 g, 5.08 mmol) and 9H-carbazole-9-ethanol (2.14 g, 10.15 mmol) were dissolved in dry DMF (10 mL) under an inert atmosphere. After the addition of Cs_2_CO_3_ (1.0 g, 3.07 mmol), the reaction content was stirred for 7 days at room temperature and then treated with an ice/water mixture. The precipitation was filtered off and purified by applying a column chromatographic technique (silica gel: stationary phase; dichloromethane: mobile phase). A white powder was obtained by evaporating the eluent. Molecular formula: C_36_H_26_N_4_O_2_. Yield: 1.03 g (37%). ^1^H NMR (500 MHz; DMSO-*d*_6_): δ 8.13–8.09 (d, 4H), 7.80 (s, 2H), 7.68–7.65 (d, 4H), 7.47–7.43 (t, 4H), 7.30–7.26 (m, 2H), 7.15 7.10 (t, 2H), 4.89–4.85 (t, 4H), 4.67–4.63 (t, 4H). ^13^C­{^1^H} NMR (126 MHz; DMSO-*d*_6_): δ 157.6, 151.3, 140.4, 135.2, 127.3, 126.0, 125.8, 122.6, 120.8, 119.5, 119.3, 118.8, 115.7, 115.5, 115.3, 110.2, 109.9, 108.2, 107.5, 107.3, 69.3, 42.0.

#### 3.1.3 Zinc Phthalocyanine

4,5-bis­(9H-carbazole-9-ethoxy)­phthalonitrile (0.100 g, 0.183 mmol), Zn­(CH_3_COO)_2_ (0.009 g, 0.046 mmol), and an extra amount of DBU were stirred in *n*-hexanol (2 mL) at 150 °C under a nitrogen atmosphere for 24 h. The reaction was carried out in an oil bath. After cooling to room temperature, the reaction content was poured into a methanol/water mixture (1:1 v/v) and filtered off. The pure product was obtained by performing column chromatography on silica gel eluted with ethyl acetate. A dark green powder was collected after evaporation of the mobile phase. Yield: (0.070 g, %68). ^1^H NMR (500 MHz; DMSO-*d*_6_): δ 8.09–8.06 (d, 16H), 7.82 (s, 8H), 7.71–7.69 (t, 16H), 7.68–7.66 (d, 16H), 7.60–7.57 (m, 16H), 4.89–4.86 (t, 16H), 4.67–4.69 (t, 16H). UV–Vis (DMSO), λ_max_, nm: 360, 692. MS (MALDI-TOF): *m*/*z* calcd for C_144_H_104_N_16_O_8_Zn [M]^+^, 2251.85; found, 1980.89 [M-2C_14_H_12_NO–5H + DHB]^+^.

#### 3.1.4 Preparation of Nanoconjugates

Unmodified gold nanoparticles (**Au20** and **Au40**) and phthalocyanine-functionalized gold nanoparticles (**Au20/SiPc, Au20/ZnPc Au40/SiPc**, and **Au40/ZnPc**) were synthesized as described in the literature with some modifications^13,21^ and their coded names are listed in Table 1.

#### 3.1.5 Gold Nanoparticles (**Au20** and **Au40**)

The aqueous solution of trisodium citrate (4.5 mL for **1** and 3.5 mL for **2**) was added to the aqueous solution of chloroauric acid, which was boiled. The mixture was stirred vigorously and stopped when the color changed. After cooling to room temperature, the solution was kept at 4 °C.

#### 3.1.6 Nanoconjugates (**Au20/SiPc, Au20/ZnPc Au40/SiPc**, and **Au40/ZnPc**)

Five mg of each macromolecule (a or b) was dissolved in a sufficient amount of dimethyl sulfoxide and added to 10 mL of the prepared gold nanoparticles. The mixture was stirred vigorously at room temperature for 18 h, centrifuged, and recollected.

### 3.2 Cells and Cell Culture

Human adenocarcinoma alveolar basal epithelial cells (A549) (#CCL-185, ATCC) were a kind gift of Dr. Rengin Reis (ACU). The cells were cultured in DMEM F12 Medium (#11320033, Gibco USA) with 10% fetal bovine serum (FBS) (Biological Industries, Israel #04–007–1A) and 0.1% penicillin–streptomycin (PS) (Gibco, USA #15140–122). Human umbilical vein endothelial cells (HUVEC) (#C2519A, Lonza Switzerland) were cultured on 0.5% gelatin coated plates with endothelial growth medium 2 (EGM-2) (#C-22111, PromoCell USA) with supplements and PS (0.1%). Media were changed every 2 days following a gentle wash of the cell monolayer with warm PBS. The cells were expanded for 4 days (37 °C in 5% CO_2_) until they reached confluence. NCs in DMSO (1 mg/mL stock) were diluted in culture media (1:100, 10 μg/mL) before applying to the cells. Morphological evaluation and imaging of the cells were conducted using an inverted microscope with Axiocam ERc5s camera in bright field mode (Zeiss Primovert, Germany).

### 3.3 Cell Viability Assay

Cell viability was assessed using the Presto Blue Assay (#A13261 ThermoScientific, USA). A549 cells were seeded in a 96-well plate (p29, 5 × 10^3^ cells/well) and maintained overnight (37 °C, 5% CO_2_). The following day, the medium was removed, and NCs were then added in replicates (*n* = 5) and incubated for 24 h. All compounds were initially dissolved in DMSO and subsequently diluted (1:1000) in culture medium to obtain final concentrations of 1 μg/mL. Control groups received the same medium without compounds, while a positive cytotoxicity control was prepared using 5% DMSO (*n* = 3). Following the 24 h incubation, the media were removed, the cells were washed with prewarmed PBS, and then 10% Presto Blue reagent (in DMEM without phenol red (#31053028 Gibco, USA)) was applied to all wells. After 1.5 h of incubation, the optical density was measured at 570 and 600 nm using a multimode plate reader (Victor Nivo 5T, PerkinElmer USA). The dye solution was then removed, and the cells were washed with prewarmed PBS before re-exposure to NCs for an additional 48 h. At the end of the total 72 h incubation period, Presto Blue reagent was added, and optical density was measured as described above. Data were processed according to the manufacturer’s guidelines. The % cell viability of control wells was set to 100% to normalize the results for the test groups.

### 3.4 Statistical Analyses

Cell viability experiments were conducted with 15 replicates per group (*n* = 3 biological replicates × 5 technical replicates). The dye solution was served as the blank for background absorbance (*n* = 3). Data are presented as mean ± standard deviation (SD). Differences between groups were evaluated by one-way analysis of variance (ANOVA) followed by Dunnett’s multiple comparisons test, with the untreated control group serving as the reference. Statistical significance was set at *p* < 0.05. All statistical tests were carried out using GraphPad Prism version 10.5.0.

## 4 Conclusions

In this study, we synthesized and fully characterized a panel of Pc–AuNP nanoconjugates varying in metal center (Si­(IV) vs Zn­(II)), substitution geometry (axial vs peripheral), and core diameter (20 nm vs 40 nm). Morphology was examined using TEM and SEM techniques. The effects of these nanoconjugates on the morphology of healthy human umbilical vein endothelial cells (HUVECs) and cancerous human lung adenocarcinoma cells (A549) were assessed by bright-field microscopy, with additional high-magnification imaging to visualize nanoparticle aggregates in and around the cells. Cell viability assessment of A549 cells by PrestoBlue assay showed a higher viability (*p* < 0.05) for nanoconjugate **Au40/SiPc** compared to all groups, suggesting potential ROS reducing effect, whereas converged to control levels at 72 h. By integrating structural variation, cell-type comparison, and methodological validation, this work aims to provide a comprehensive assessment of structurally distinct Pc–AuNP nanoconjugates and highlight the importance of assay selection when evaluating nanoparticle-based photosensitizers in both healthy and cancer cell models.

## Supplementary Material



## Data Availability

The data underlying this study are available in the published article and its Supporting Information.

## Notes

The following Supporting Information can be downloaded at: **S1:** ^1^H NMR (500 MHz; DMSO-*d*_6_) spectrum of macromolecule (**SiPc**), **S2:** UV–vis spectrum of macromolecule (**SiPc**) in DMSO, **S3:** MALDI-TOF spectrum of macromolecule (**SiPc**), **S4:** ^1^H NMR (500 MHz; DMSO-*d*_6_) spectrum of the phthalonitrile derivative, **S5:** ^13^C­{^1^H} NMR (126 MHz; DMSO-*d*_6_) spectrum of the phthalonitrile derivative, **S6:** ^1^H NMR (500 MHz; DMSO-*d*_6_) spectrum of macromolecule (**ZnPc**), **S7:** UV–vis spectrum of macromolecule (**ZnPc**) in DMSO, **S8:** MS (MALDI-TOF) spectrum of macromolecule (**ZnPc**), **S9:** FT-IR spectra of unmodified (**Au20** and **Au40**) and modified gold nanoparticles (**Au20/SiPc**, **Au20/ZnPc**, **Au40/SiPc**, and **Au40/ZnPc**), **S10:** UV–vis spectra of **SiPc**, **ZnPc**, unmodified gold nanoparticles (**Au20** and **Au40**), and nanoconjugates (**Au20/SiPc**, **Au20/ZnPc**, **Au40/SiPc**, and **Au40/ZnPc**) in 1% DMSO at room temperature (PDF)

## Abbreviations

TEM: transmission electron microscopy
SEM: scanning electron microscopy
FT-IR: Fourier transform infrared spectroscopy
NMR: nuclear magnetic resonance
