# Supplementary material for: Aggregation Tendency, Cellular Uptake, and Viability Effects of Structurally Distinct Carbazole–Phthalocyanine Gold Nanoconjugates
Source: ACS Org Inorg Au. 2026 Jan 26;6(1):149–60. doi: 10.1021/acsorginorgau.5c00107 (PMC12879172; doi:10.1021/acsorginorgau.5c00107)
Supplement: Supplementary file 1 [file gg5c00107_si_001.pdf]

Aggregation Tendency, Cellular Uptake, and Viability Effects of Structurally Distinct Carbazole–Phthalocyanine Gold Nanoconjugates

Neval Sevinç Özdemir [1,2], Özlem İpsiz Öney [3], Hacer Yasemin Yenilmez [3], Nazlı Farajzadeh Öztürk[4] and Zehra Altuntaş Bayır [3,*]

[1] Department of Pharmaceutical Basic Sciences, Faculty of Pharmacy, Acıbadem Mehmet Ali Aydınlar
University, Ataşehir, Istanbul, 34752, Türkiye (email: neval.sevinc@acibadem.edu.tr)

[2] ACU Biomaterials Center, Acıbadem Mehmet Ali Aydınlar University, 34752 Istanbul, Türkiye

[3] Department of Chemistry, Istanbul Technical University, Maslak, Istanbul, 34469, Türkiye (email: ipsiz@ itu.edu.tr; yenilmez@itu.edu.tr)

[4] Department of Analytical Chemistry, Faculty of Pharmacy, Acibadem Mehmet Ali Aydınlar University, Ataşehir, Istanbul, 34752, Türkiye (email: nazli.ozturk@acibadem.edu.tr)

* Correspondence: bayir@itu.edu.tr

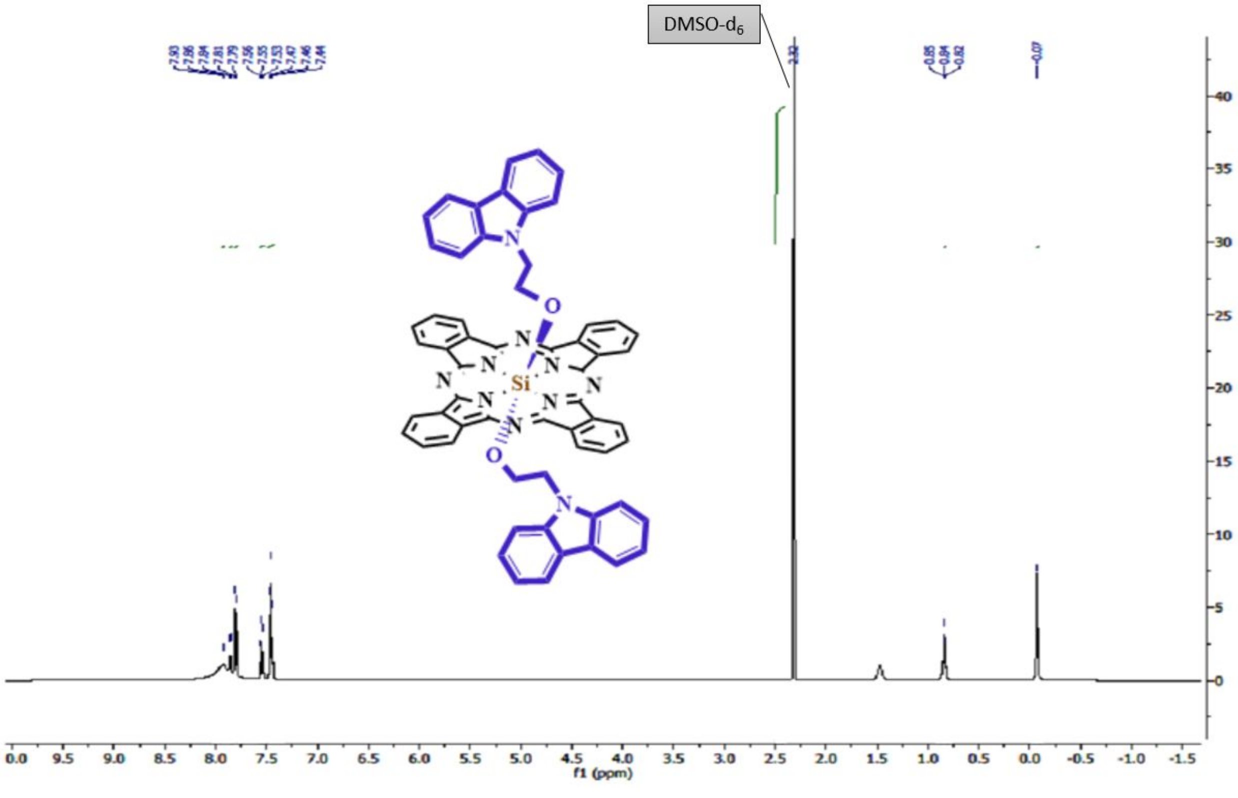

S1. ^{1}H NMR (500 MHz; DMSO-d_6) spectrum of macromolecule (**SiPc**).

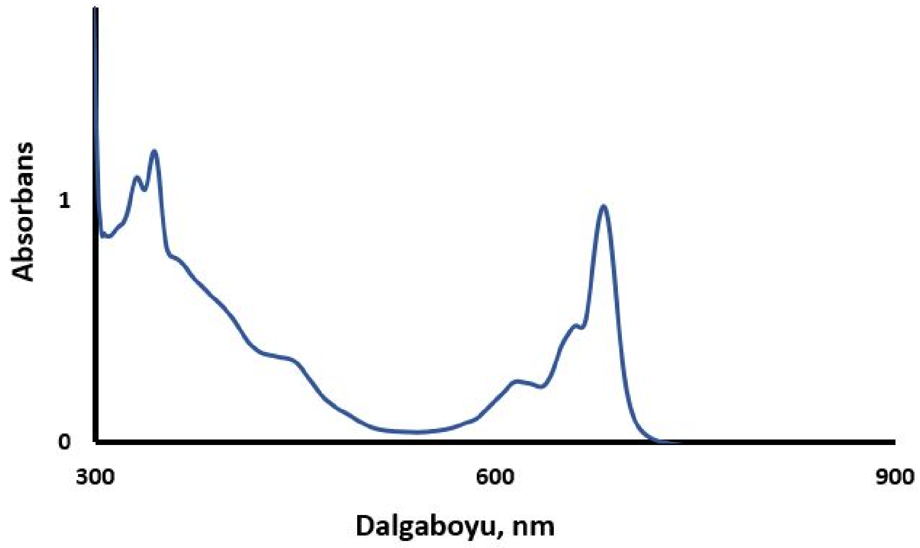

S2. UV-vis spectrum of macromolecule (**SiPc**) in DMSO.

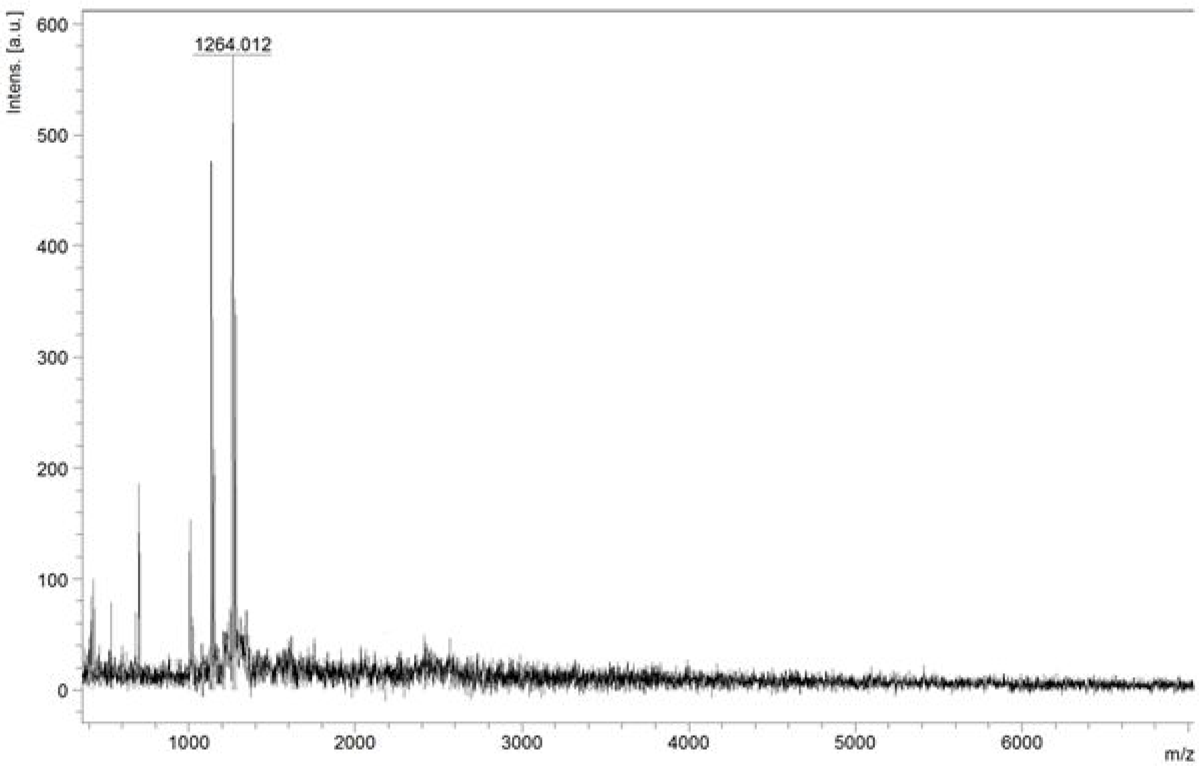

S3. MALDI-TOF spectrum of macromolecule (**SiPc**).

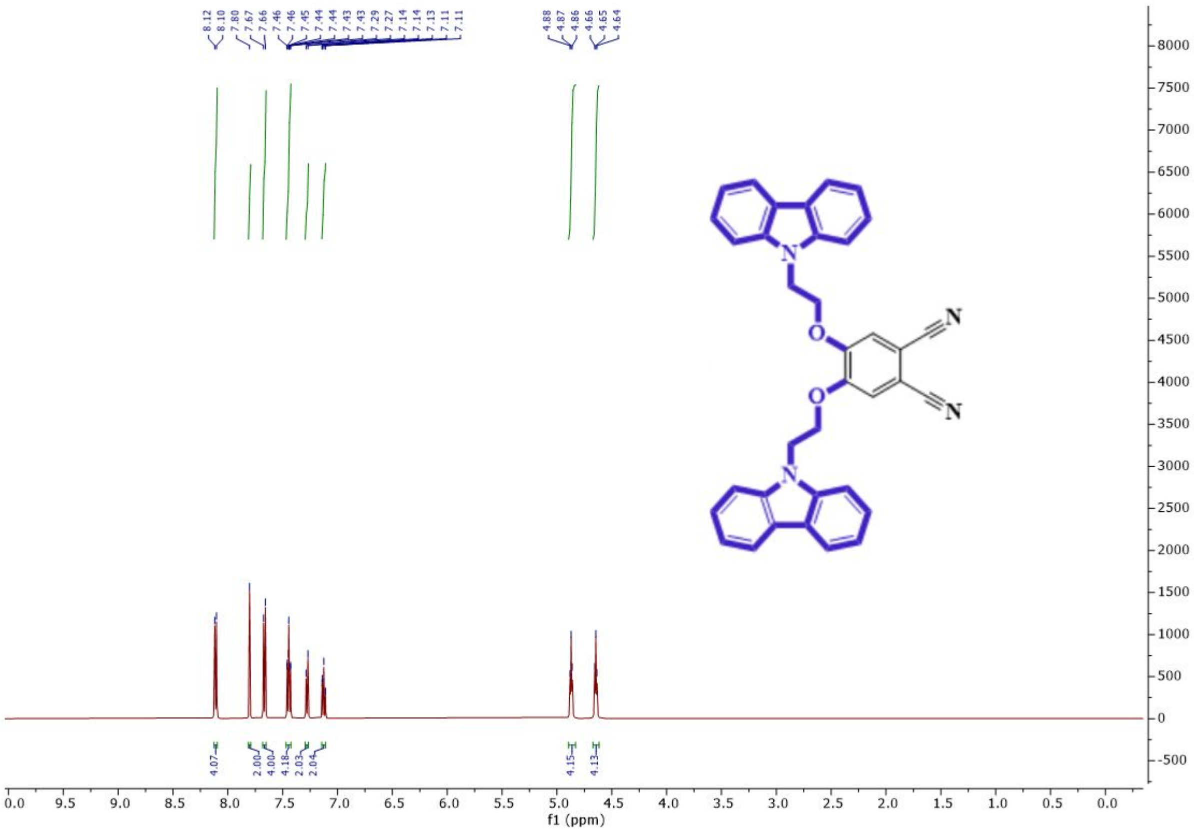

S4. ^{1}H NMR (500 MHz; DMSO-d_6) spectrum of the phthalonitrile derivative.

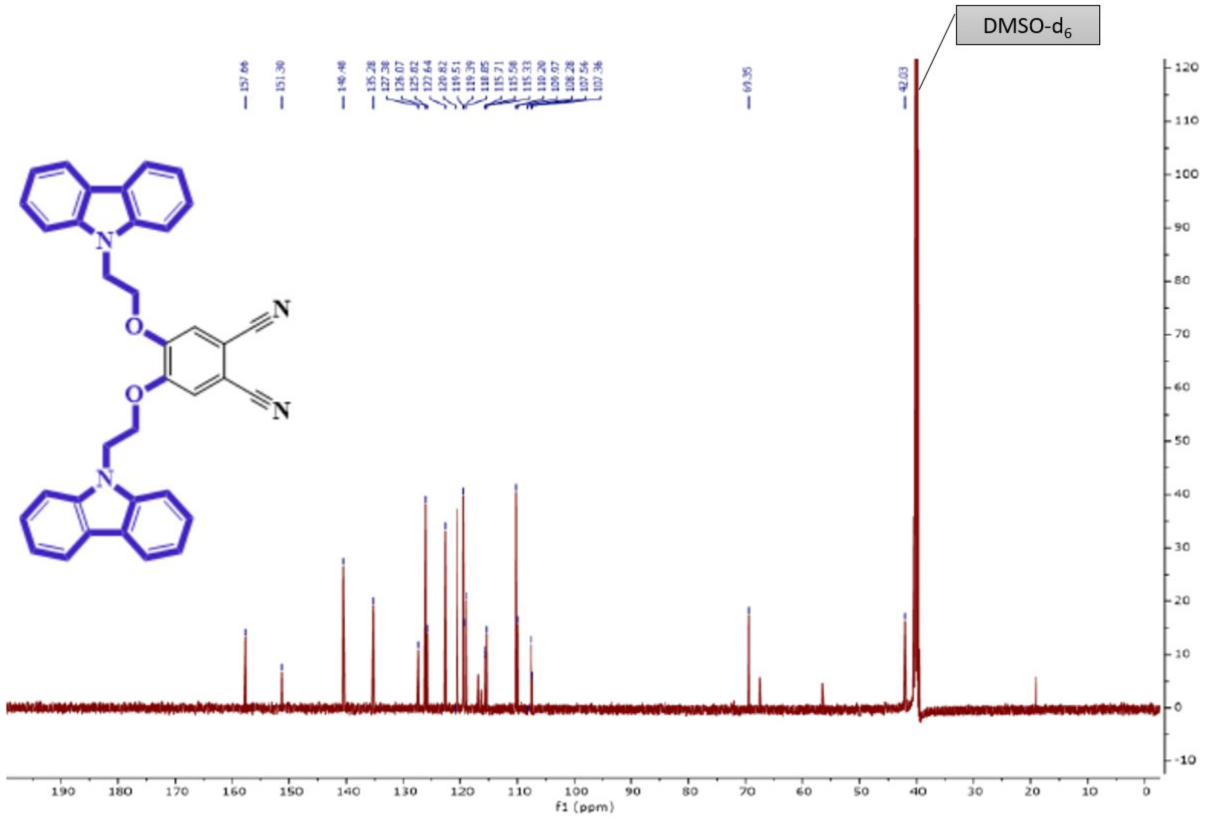

S5. $^{13}C\{^{1}H\}$ NMR (126 MHz; DMSO-d_6) spectrum of the phthalonitrile derivative.

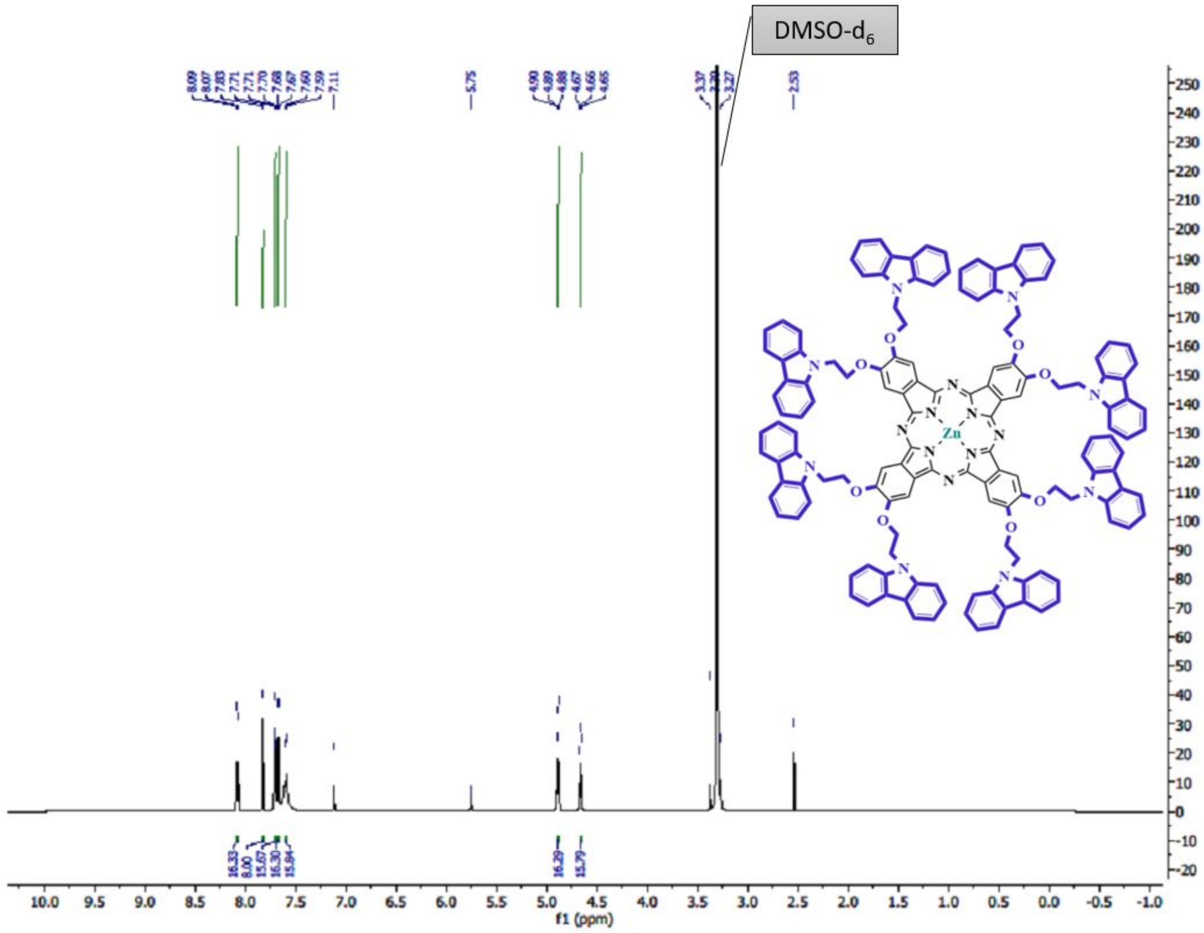

S6. ^{1}H NMR (500 MHz; DMSO-d_6) spectrum of macromolecule (**ZnPc**).

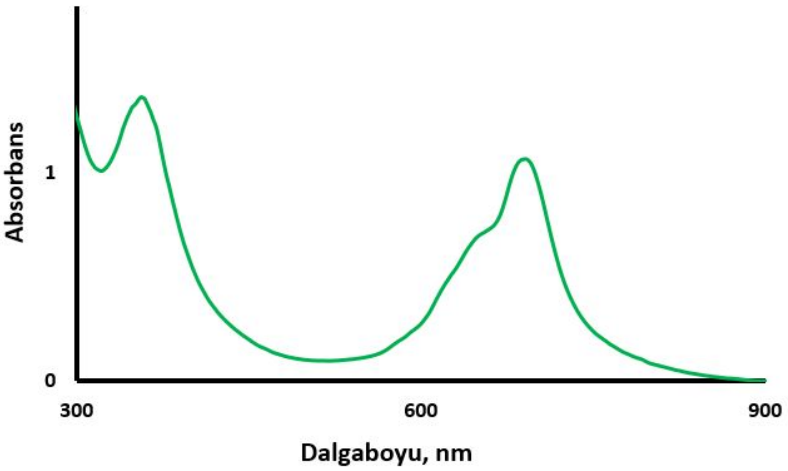

S7. UV-vis spectrum of macromolecule (**ZnPc**) in DMSO.

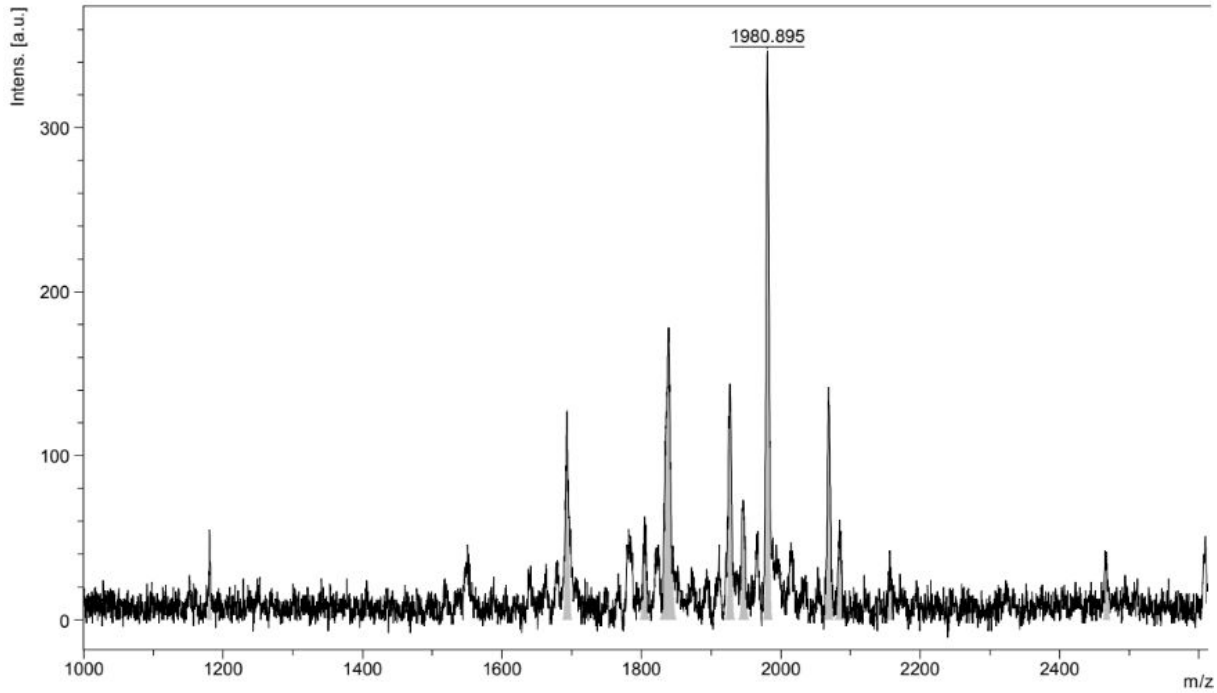

S8. MS (MALDI-TOF) spectrum of macromolecule (**ZnPc**).

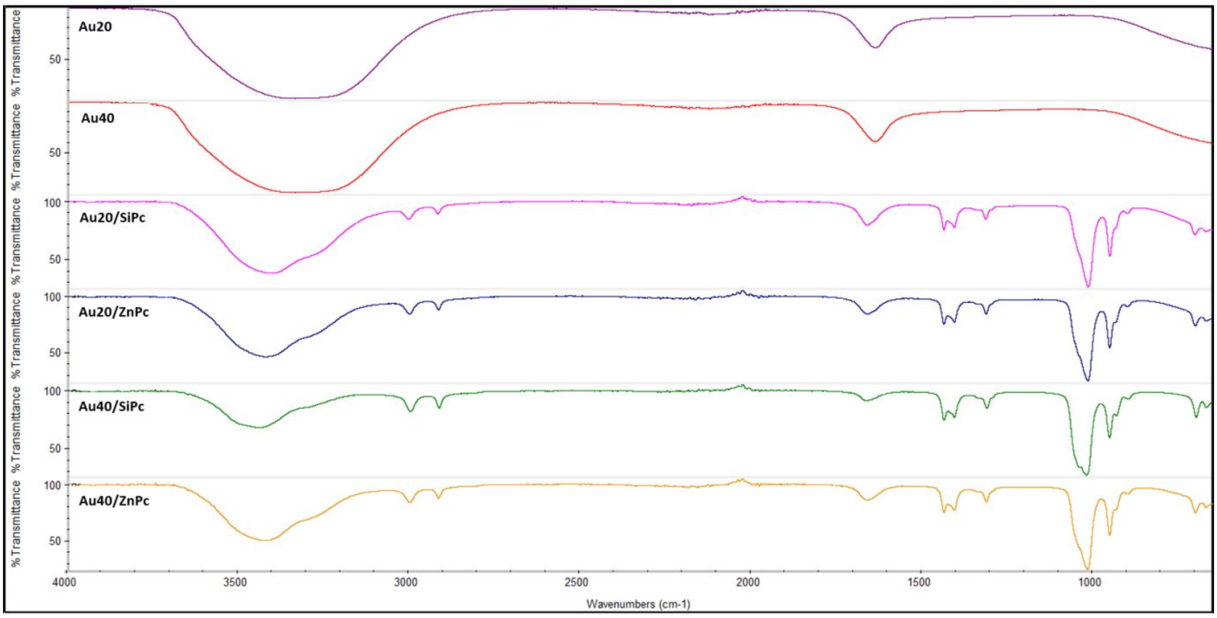

S9. FT-IR spectra of unmodified (**Au20** and **Au40**) and modified gold nanoparticles (**Au20/SiPc**, **Au20/ZnPc**, **Au40/SiPc**, and **Au40/ZnPc**).

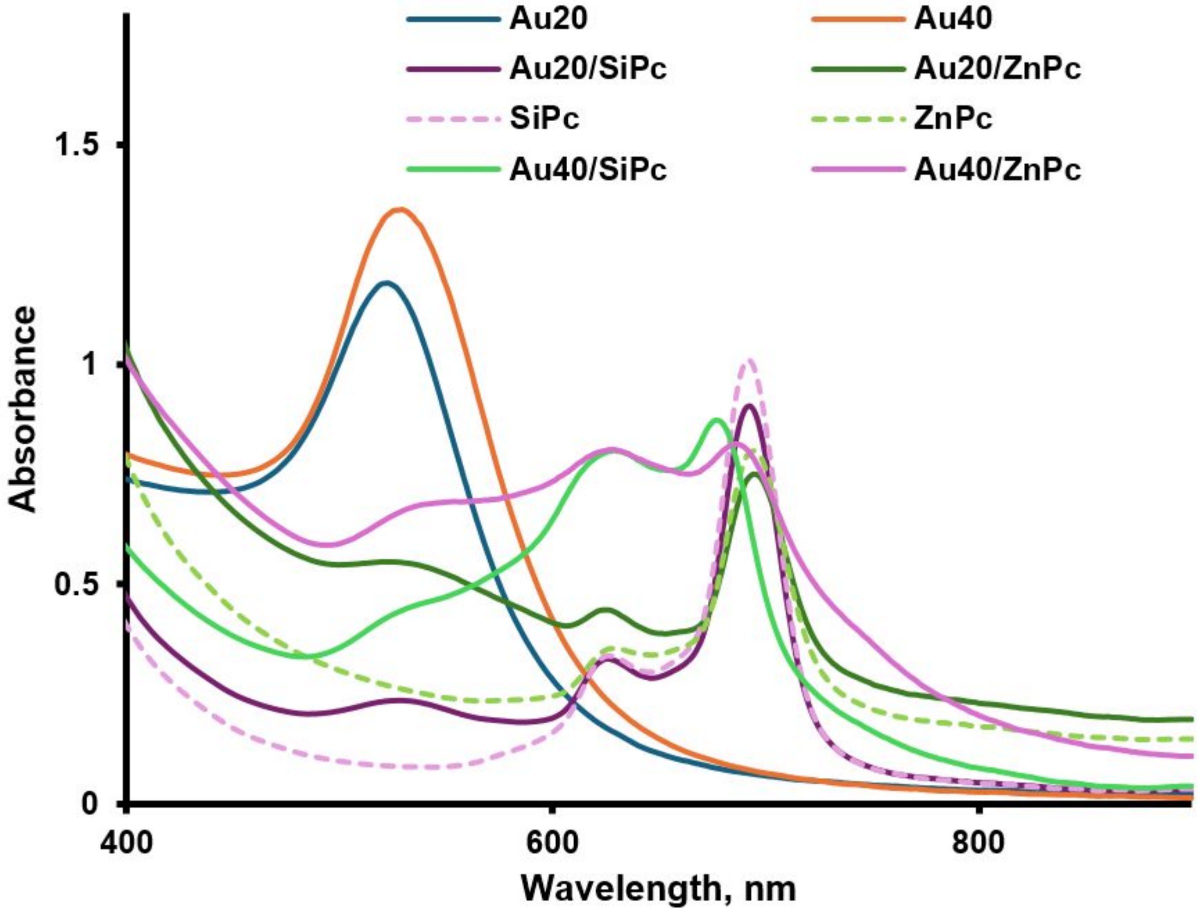

S10. UV-vis spectra of **SiPc**, **ZnPc**, unmodified gold nanoparticles (**Au20** and **Au40**), and nanoconjugates (**Au20/SiPc**, **Au20/ZnPc**, **Au40/SiPc**, and **Au40/ZnPc**) in 1%DMSO at room temperature.

.
